# Plasma androgens and the presence and course of depression in a large cohort of women

**DOI:** 10.1038/s41398-021-01249-2

**Published:** 2021-02-12

**Authors:** Anouk E. de Wit, Erik J. Giltay, Marrit K. de Boer, Fokko J. Bosker, Aviva Y. Cohn, Willem A. Nolen, Ursula B. Kaiser, Hadine Joffe, Brenda W.J.H. Penninx, Robert A. Schoevers

**Affiliations:** 1 University of Groningen, University Medical Center Groningen, Department of Psychiatry, Groningen, The Netherlands; 2 Leiden University Medical Center, Department of Psychiatry, Leiden, The Netherlands; 3 Brigham and Women’s Hospital, Harvard Medical School, Department of Medicine, Boston, MA USA; 4 Brigham and Women’s Hospital, Harvard Medical School, Department of Psychiatry, Boston, MA USA; 5 Brigham and Women’s Hospital, Harvard Medical School, Connors Center for Women’s Health and Gender Biology, Boston, MA USA; 6 Department of Psychiatry, Amsterdam UMC, VU University, Amsterdam, The Netherlands

**Keywords:** Diagnostic markers, Prognostic markers, Depression, Predictive markers

## Abstract

Major depressive disorder (MDD) has a higher prevalence in women with supraphysiologic androgen levels. Whether there is also an association between depression and androgen levels in the physiological range, is unknown. This study examined if women with current MDD have higher androgen levels compared to women who have never had MDD, and if androgen levels are associated with onset and remission of MDD. In 1659 women (513 current MDD, 754 remitted MDD, and 392 never MDD), baseline plasma levels of total testosterone, 5α-dihydrotestosterone, and androstenedione were determined with liquid chromatography-tandem mass spectrometry, and dehydroepiandrosterone-sulfate and sex hormone binding globulin (SHBG) with radioimmunoassays. Free testosterone was calculated. MDD status was assessed at baseline, and at 2 and 4 years follow-up. Women were aged between 18 and 65 years (mean age 41) with total testosterone levels in the physiological range (geometric mean 0.72 nmol/L [95% CI 0.27–1.93]). After adjusting for covariates and multiple testing, women with current MDD had a higher mean free testosterone than women who never had MDD (adjusted geometric mean 8.50 vs. 7.55 pmol/L, *p* = 0.0005), but this difference was not large enough to be considered clinically meaningful as it was consistent with statistical equivalence. Levels of other androgens and SHBG did not differ and were also statistically equivalent between the groups. None of the androgens or SHBG levels predicted onset or remission of MDD. Our findings support the idea that plasma androgens within the physiological range have no or only limited effects on depressive disorders in women.

## Introduction

Ever since the recognition of the greater preponderance of major depressive disorder (MDD) in women compared to men^1^, sex steroids, like estrogens and also androgens, have been proposed to play a role in mood. Amongst women, the prevalence of MDD is further increased in patients with medical conditions that are associated with elevated androgen levels, such as polycystic ovarian syndrome and congenital adrenal hyperplasia^2,3^. This suggests that circulating androgens in women may have detrimental effects on mood. Such effects may result from androgens binding to intracellular sex steroid receptors in structures of the limbic system^4^. However, systemic androgen levels may also be affected by MDD, rather than being a causal agent. Hyperactivity of the HPA axis in patients with MDD could stimulate the adrenal cortex to not only enhance the release of cortisol, but of adrenal androgens as well^5,6^.

Epidemiological studies that have examined androgens (e.g., total testosterone) in relation to MDD prevalence, which is the product of incidence and episode duration^7^, have shown equivocal results. Three cross-sectional studies found that women with current MDD had higher levels of total testosterone compared to non-depressed controls^8–10^, whereas another study reported the opposite^11^, and two other studies reported no differences^12,13^. Four large prospective studies examined whether total testosterone levels predicted the incidence of MDD up to 10 years after baseline in adult women from the general population^8,14–16^. The majority reported no link^14–16^, whereas one study showed that an annual increase in total testosterone was associated with increased risk of MDD in women during perimenopause^17^. Only one study examined whether testosterone levels predicted the duration of MDD in 297 older women; those with lower plasma levels of total and free testosterone tended to have a higher severity of depression during 2 years follow-up that approached statistical significance^18^.

The inconsistencies in these findings highlight the need to re-address the question about the role of androgens in MDD in adult women. This research should overcome previous limitations that include: (1) the use of screening tools for diagnosing MDD rather than structured diagnostic interviews, (2) failure to adjust for oral contraceptive and hormonal replacement therapy use, and (3) the determination of androgens by immunoassays rather than liquid chromatography-tandem mass spectrometry (LC-MS/MS)^19^. Moreover, analyses should include other androgens besides free and total testosterone, as many additional endogenous androgenic compounds exist.

Here we sought to examine the role of plasma androgens in MDD in women. We hypothesized that: (1) androgen levels are higher in women with current MDD compared to women who never had MDD, (2) in women without current MDD, there is an elevated risk for a new episode with higher androgen levels, and (3) in women with current MDD, higher levels of androgens are associated with a longer time to recovery. Looking at a cohort of women from the clinically well-phenotyped Netherlands Study of Depression and Anxiety (NESDA), multiple androgens were measured by LC-MS/MS. These androgen levels were examined in relation to the presence and course of MDD over 4 years, while taking into account important confounders such as age and oral contraceptive use.

## Materials and methods

### Study population

Data were derived from NESDA; a large multi-center study designed to investigate the long-term course and consequences of depressive and anxiety disorders. Between 2004 and 2007, 2981 currently and former depressed and anxious, and euthymic men and women (aged 18–65 years) were recruited. Exclusion criteria included not speaking Dutch fluently, or being diagnosed with one of the following psychiatric diseases according to DSM-IV criteria: psychotic disorder, obsessive compulsive disorder, bipolar disorder, or substance dependence^20^. The ethical committees of the participating centers approved the study design and all participants gave verbal and written informed consent.

For this study, 1979 women were included. Exclusion criteria included women who had missing plasma androgen measurements (*n* = 40) or extreme androgen values (>8 SD below or above the mean, *n* = 5), and women who were pregnant (*n* = 17) or transgender (*n* = 1). Women who used androgens (World Health Organization Anatomical Therapeutic Chemical (ATC) code^21^ G03B and E), gonadotrophins (ATC code G03G), antiandrogens (ATC code G03H), gonadotrophin-releasing hormone analogs (ATC code L02AE), antiandrogens (ATC code L02BB), or aromatase inhibitors (L02BG) during the blood draw at baseline (*n* = 13) were also excluded. Finally, women who were diagnosed (currently or in the past) with anxiety disorder but not with MDD (*n* = 320) were excluded as well. The final baseline sample comprised 1659 women (83.8% of all women). Of this baseline sample, 1432 women (85.3%) had participated at least once in a NESDA follow-up assessment after 2 or 4 years. Those without follow-up assessments (*n* = 227, 12.2%) had received less education (mean ± SD years of education 11.3 ± 3.1 vs. 12.4 ± 3.2, *p* < 0.001), and more often suffered from MDD (68.5% vs. 44.4%, *p* < 0.001) and/or an anxiety disorder (47.8% vs. 25.2%; Supplement 1).

### Hormone assays

Androgens were measured in plasma. Blood draws took place in the morning (mean time 08:48 h SD 22 min) after an overnight fast (success rate 96.1%). Total testosterone, 5α-dihydrotestosterone (5α-DHT), and androstenedione were determined by LC-MS/MS at the Clinical Chemistry department of the University Medical Centre Groningen. The lower limit of quantitation was 0.04 nmol/L for total testosterone and androstenedione, and 0.12 nmol/L for 5α-DHT. Women who had values below the detection limit for 5α-DHT (*n* = 153, 9.2%) were given values 0.01 nmol/L less than the lower limit of quantitation. Dehydroepiandrosterone-sulfate (DHEAS) and sex hormone binding globulin (SHBG) were determined using ARCHITECT (ABBOTT, Wiesbaden, Germany), a one and two-step radioimmunoassay with chemiflex assay protocols. The lower limit of detection of DHEAS was ≤0.3 μmol/L with a calibration range of 0.00–40.71 μmol/L, and ≤0.1 nmol/L for SHBG with a calibration range of 0.0–250 nmol/L. The inter-assay coefficients of variation were estimated to be ≤10% for both. Free testosterone was calculated based on SHBG and total testosterone levels, and an assumed albumin level of 43 g/L with the formula of Vermeulen^22^. As a measure of HPA-axis activation, salivary cortisol levels were determined. These measures were used to calculate the area under the curve (AUC) with respect to the ground (AUC_g_) or increase (AUC_i_) of salivary cortisol. For more details on the determination of cortisol and the calculation of the AUC we refer to previous work^23^. In brief, saliva samples were collected at baseline using Salivettes (Sarstedt AG and Co, Nümbrecht, Germany) at awakening, and 30, 45, and 60 min later. After the determination of cortisol levels using competitive electrochemiluminescence immunoassay (E170; Roche, Basel, Switzerland), the AUC_g_ and AUC_i_ was calculated. Missing values on this covariate (*n* = 655, 39.5%) were not imputed, as substitution of this large amount of data would cause bias.

### Presence, remission, and onset of major depressive disorder

At baseline, women were categorized into three groups according to their psychopathology status: never MDD (no current or past psychopathology, *n* = 392), remitted MDD (history of MDD, but currently not depressed, *n* = 754), and current MDD (currently depressed, *n* = 513). The presence of MDD, currently or in the past, was ascertained using the lifetime version of the Composite International Diagnostic Interview (CIDI [version 2.1])^24^. A current MDD was ascertained when women fulfilled the Diagnostic and Statistical Manual of Mental Disorders (DSM)-IV-based CIDI criteria within the past month, and remitted MDD was determined when participants had MDD earlier in life, but not within the last month. The CIDI is a validated instrument with high inter-rater reliability (any depressive disorder *κ* = 0.95) and high validity for depressive and anxiety disorders^24,25^. The severity of depressive symptoms were assessed with the 30-item self-report Inventory of Depressive Symptoms^26^. Time to remission was determined as the number of years until a participant no longer fulfilled the criteria for MDD in the subset of depressed participants at baseline on the CIDI. First or recurrent onset of MDD on the other hand, was ascertained when women without MDD at baseline fulfilled the criteria of MDD during follow-up. For both outcomes, participants who dropped out before the first follow-up assessment (*n* = 108 for time to remission, and *n* = 119 for time to onset) were excluded, and dropouts after that point were censored. This resulted in samples of 405 and 1027 participants who were used for calculating time to remission, and time to onset of MDD, respectively.

### Statistics

At baseline, group characteristics of never MDD, current MDD, and remitted MDD were compared using one-way analysis of variance (ANOVA) for independent samples or *χ*2 tests, depending on whether the variable was continuous or categorical. Due to right-skewed distributions, all androgen levels were log_e_-transformed, and to ease comparability of effect sizes, standardized before the analyses (into *z*-values).

For the first hypothesis, we compared group means of androgen levels of women with never MDD to those with remitted and current MDD, using two-way analyses of covariance (ANCOVA). Analyses were conducted with and without adjustment for the following time-independent covariates measured at baseline; age (in years), education (in years), smoking status (dichotomously: smoker/no smoker), alcohol use (dichotomously: ≤ or >7 units a week), measured body mass index (BMI) (continuously), number of treated chronic somatic disorders (ordinal), oral contraceptive use (dichotomously: use/ no use), hormonal replacement therapy use (dichotomously: use/ no use), and menopausal status (dichotomously: postmenopausal / not postmenopausal). The definition of the covariates and the motivation for their use, are described in Supplement 2. Missing values on menopausal status were imputed with postmenopausal when women were aged ≥51 years (*n* = 21; 1.2%). Missing values on the covariate alcohol use (*n* = 24; 1.4%) were imputed with the mean. Other covariates had no missing values. Results were back-transformed to the original scale to get the geometric mean with 95% confidence interval (CI) for visualization purposes. Using linear regression analysis, we additionally examined whether the potential association between androgen levels and MDD is driven by the severity on a continuous scale rather than the dichotomous diagnostic categorization. As a sensitivity analysis, we examined whether the association between androgen levels and severity of depressive symptoms was different between pre- and postmenopausal women by adding the interaction “androgen level*menopausal status” to the analyses. As a second sensitivity analyses, we examined whether the associations were better described by a non-linear relationship by adding quadratic terms of androgen levels to the analyses.

For the second and third hypotheses, we analyzed the association of androgen levels to both time to onset and time to remission of MDD using Cox proportional hazard models. We checked whether the analyses met the Cox proportional hazards assumption with appropriate log-minus-log (LML) curves. Again, models were adjusted for the covariates measured previously, and additionally for lifetime MDD or anxiety (time to first or recurrent MDD), or for lifetime anxiety disorder (time to remission of MDD). In sensitivity analyses, non-linear associations were examined by adding squared androgen levels to the models.

Finally, we explored whether altered levels of androgens in women with MDD, if present, might be mediated by salivary cortisol (AUC_g_ and AUC_i_) using the indirect method by Preacher and Hayes^27^. This method estimates the total, direct, and indirect effects of the independent variable on the dependent variable through the mediator variable.

Data were analyzed using IBM SPSS Statistics (IBM Corp) version 25, using two-sided tests. As multiple tests were performed, we calculated an adjusted false discovery rate *p* cut-off value to avoid the inflation of false-positive findings. A *p*-value of < 0.0075 was considered statistically significant.

## Results

The baseline characteristics of the 1659 women are shown in Table 1. Women who suffered from current MDD, followed successively by women with remitted MDD and never MDD, presented with the highest mean BMI scores (26.0, 25.1, and 24.7, *p* < 0.001), the highest mean number of chronic diseases (0.6, 0.5, and 0.4, *p* < 0.001), and the highest percentage of current smokers (45.4%, 39.0%, and 24.0%, *p* < 0.001). Levels of total testosterone, free testosterone, 5α-DHT, androstenedione and DHEAS were lower, while SHBG was elevated in oral contraceptive users compared to non-users. See Supplement 3.

**Table 1:** Characteristics of 1659 women at baseline.

	Never MDD (*n* = 392)^a^	Remitted MDD (*n* = 754)^a^	Current MDD (*n* = 513)^a^	*p*
Age, mean ± SD	40.7 ± 14.4	41.4 ± 12.7	40.6 ± 12.2	0.49
Education, mean ± SD	12.7 ± 3.2	12.5 ± 3.2	11.4 ± 3.2	<0.0001
Body mass index, mean ± SD	24.7 ± 4.7	25.1 ± 5.1	26.0 ± 5.9	<0.0001
Smoking, no. (%)	94 (24.0)	294 (39.0)	233 (45.4)	<0.0001
**>**1 alcohol unit/day, no. (%)	52 (13.3)	101 (13.4)	76 (14.8)	0.73
Treated chronic diseases, geo. mean (95% CI)	0.4 (0.0–1.9)	0.5 (0.0–2.3)	0.6 (0.0–2.7)	<0.0001
Oral contraceptive use, no. (%)	123 (31.4)	192 (25.5)	137 (26.7)	0.10
Hormonal replacement therapy use, no. (%)	7 (1.8)	16 (2.1)	5 (1.0)	0.02
Menopausal, no. (%)	141 (36.0)	266 (35.3)	145 (27.7)	0.02
Typical cycle length, mean ± SD^b^	30 ± 12	28 ± 4	28 ± 4	0.16
Days since last day of menstruation, mean ± SD^b^	20 ± 40	24 ± 68	30 ± 77	0.40
Antidepressant use, no. (%)	5 (1.3)	212 (28.1)	225 (43.9)	<0.0001
Current anxiety disorder, no. (%)	0 (0.0)	273 (36.2)	350 (68.3)	<0.0001
Severity of depressive symptoms (IDS), geo. mean (95% CI)	8.3 (0.0–26.1)	18.2 (2.8–46.5)	34.8 (17.1–62.2)	<0.0001

*ANOVA* analysis of variance, *IDS* Inventory of Depressive Symptomatology, *MDD* major depressive disorder.

^a^Based on one-way ANOVA for independent samples or *χ*2 tests.

^b^In subsample of premenopausal women not using oral contraceptives (*n* = 670).

### Cross-sectional associations

**Fig. 1:**
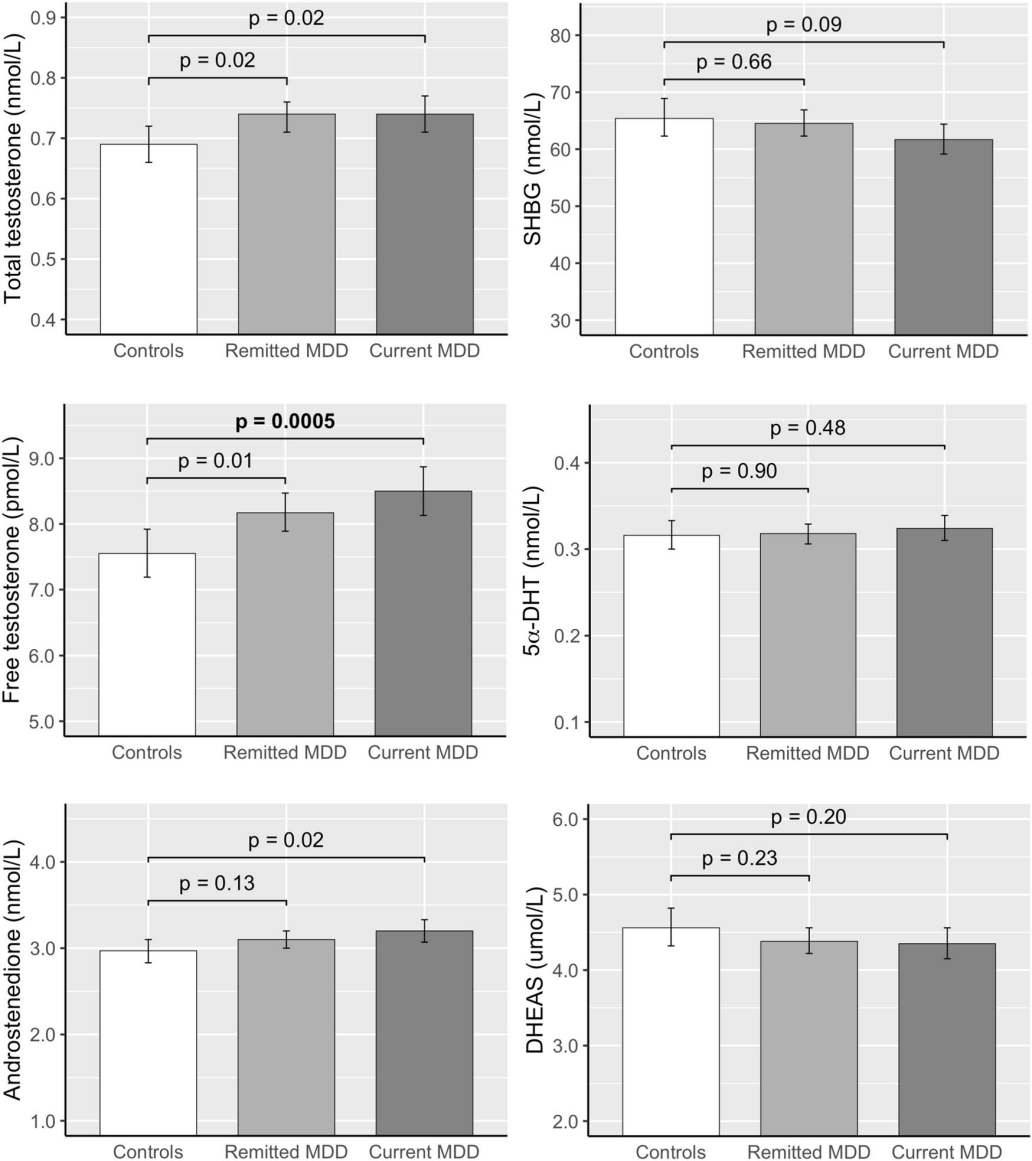
Mean androgen and SHBG levels in women according to their diagnosis at baseline. Abbreviations: 5a-DHT, 5α-dihydrotestosterone; DHEAS, dehydroepiandrosterone-sulfate. Data are geometric means with 95% CI. *p*-values are based on adjusted mean analyses by ANCOVA models. Models were adjusted for age, education, BMI, smoking, alcohol use, number of treated chronic diseases, menopausal status, oral contraceptive use, and hormonal replacement therapy use.

The (adjusted) mean plasma levels of androgens per group are depicted in Fig. 1 (see detailed ANCOVA results in Supplement 4, and raw data points as well as data distribution in Supplement 5). After adjusting for covariates, only free-testosterone levels in women with current MDD were significantly higher (by 12.6%) compared to those with never MDD (adjusted geometric mean never and current MDD: 7.55 and 8.50). Levels of SHBG, 5α-DHT, androstenedione and DHEAS did not differ between the groups. All covariates except education, were associated with at least one of the androgen or SHBG levels, but oral contraceptive use and age explained most of the variance. See the table in Supplement 2 for the *F*-statistics and *p*-values of the association of each covariate with each of the androgen and SHBG levels.

**Fig. 2:**
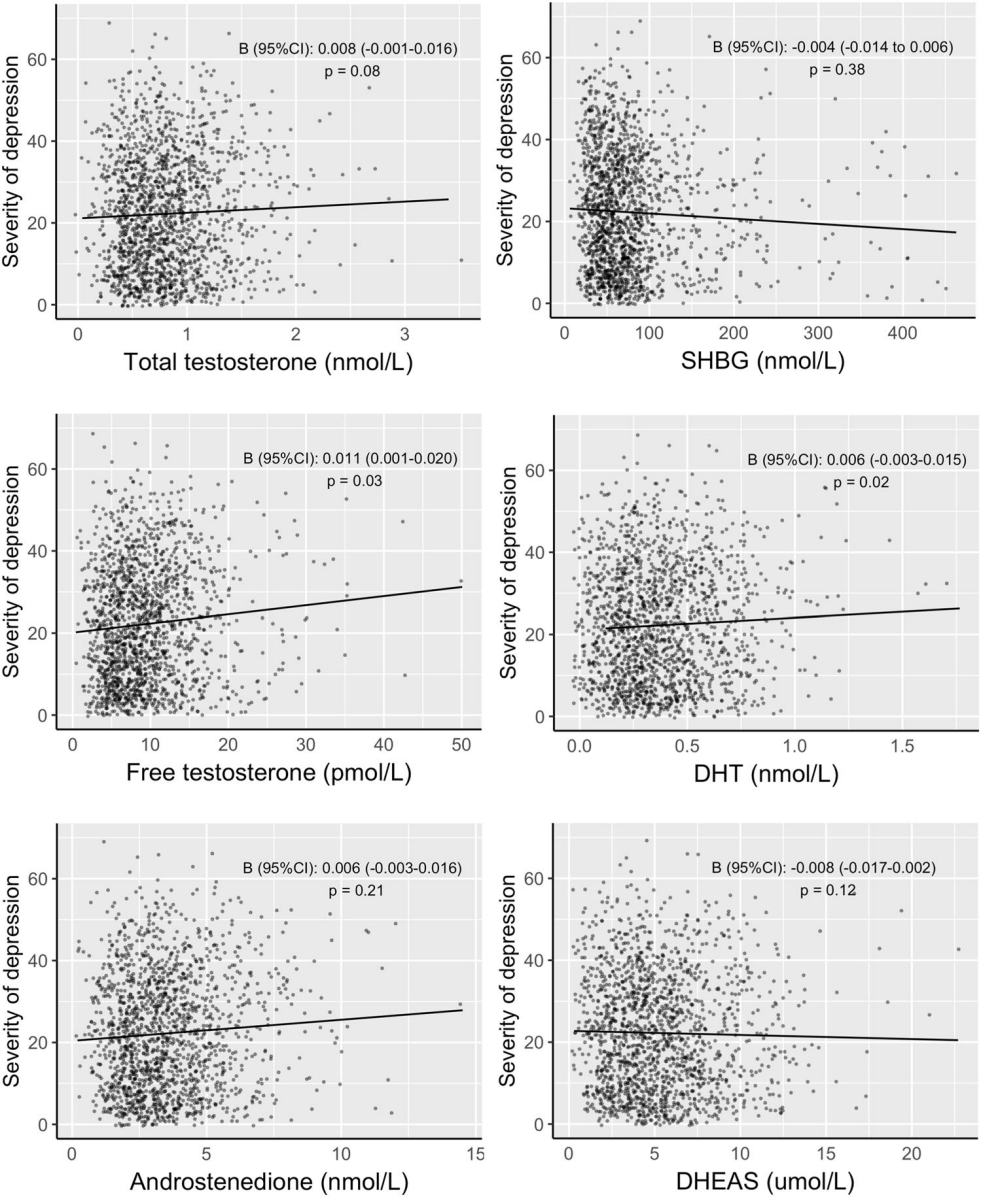
Plasma androgen and SHBG levels in women according to the severity of depression. Abbreviations: 5α-DHT, 5α-dihydrotestosterone; DHEAS, dehydroepiandrosterone-sulfate. Figure shows adjusted standardized β’s for the association between androgen levels and the depressive symptom severity scores, examined with linear regression analysis. Analyses were adjusted for age, education, BMI, smoking, alcohol use, number of treated chronic diseases, menopausal status, oral contraceptive use, and hormonal replacement therapy use. Depressive symptom severity score as measured with the Inventory of Depressive Symptomatology. To convert nmol/L to ng/dL for total testosterone, 5α-DHT, and androstenedione, multiply by 28.84, 29.07, and 28.64, respectively. To convert pmol/L to pg/mL for free testosterone, divide by 3.47. To convert µmol/L to µg/dL for DHEAS, multiply by 36.85. To convert to nmol/L to μg/mL for SHBG, multiply by 0.095.

As shown in Fig. 2 (data in Supplement 6), none of the androgens or SHBG were significantly associated with severity of depression. There was also no difference in the strength of the associations between pre- and postmenopausal women (adjusted *p*-value for the interaction of menopausal status with total testosterone *p* = 0.80, SHBG, *p* = 0.89, free testosterone *p* = 0.54, 5α-DHT *p* = 0.47, androstenedione *p* = 0.34, and DHEAS *p* = 0.81). Non-linear associations (through quadratic terms) were also all non-significant.

### Post hoc analyses

Given the generally negative findings, we used a two one-sided tests (TOST) procedure to test for equivalence and reject the presence of a smallest effect size of interest (SESOI). The TOST procedure examines whether the hypothesis that there are effects extreme enough to be considered meaningful can be rejected^28^. As to the best of our knowledge, no SESOI is known for androgens in women with regards to depression, and we used 0.5 * the SD of the baseline sample as a proxy^29^. These analyses showed that all mean differences between women with current or remitted MDD, and women who never had a MDD, were statistically equivalent. See Supplement 7, for the assumed SESOI values and results of the equivalence testing.

### Prospective associations

Table 2 shows the prospective association of androgen levels with incidence and remission of MDD, respectively. During 4 years of follow-up, 360 women out of 1027 women with no MDD at baseline (35.1%) experienced a first or recurrent episode of MDD. None of the androgens predicted time to incident MDD. Non-linear associations (through quadratic terms) were also non-significant. Conversely, MDD episodes remitted during follow-up in 254 women out of 405 women with MDD at baseline (62.7%). Again, none of the androgens predicted time to remission of MDD and non-linear associations (through quadratic terms) were also non-significant.

**Table 2:** Unadjusted and adjusted hazard ratios for time to new/recurrent episode of MDD and time to remission of MDD in women according to plasma androgen and SHBG levels during up to 4 years of follow-up.

	First or recurrent MDD		Remission MDD	
	HR (95% CI)	*p*	HR (95% CI)	*p*
Events	360 / 1027 (35.1%)		254 / 405 (62.7%)	
Total testosterone				
Unadjusted	1.14 (1.02–1.26)	0.02	1.05 (0.93–1.18)	0.48
Adjusted^a^	1.08 (0.97–1.21)	0.17	1.05 (0.92–1.20)	0.49
SHBG				
Unadjusted	0.98 (0.89–1.09)	0.75	1.01 (0.89–1.14)	0.92
Adjusted^a^	1.04 (0.91–1.12)	0.55	1.00 (0.86–1.16)	0.98
Free testosterone				
Unadjusted	1.12 (1.01–1.25)	0.03	1.03 (0.91–1.16)	0.65
Adjusted^a^	1.05 (0.93–1.19)	0.40	1.04 (0.90–1.21)	0.59
5α-DHT				
Unadjusted	1.05 (0.94–1.17)	0.38	1.05 (0.94–1.17)	0.43
Adjusted^a^	1.04 (0.92–1.17)	0.55	1.04 (0.91–1.17)	0.58
Androstenedione				
Unadjusted	1.15 (1.03–1.29)	0.01	1.07 (0.94–1.20)	0.31
Adjusted^a^	1.11 (0.98–1.25)	0.11	1.06 (0.92–1.23)	0.41
DHEAS				
Unadjusted	1.06 (0.95–1.18)	0.31	1.09 (0.97–1.22)	0.14
Adjusted^a^	1.06 (0.93–1.20)	0.37	1.10 (0.95–1.26)	0.20

*MDD* major depressive disorder, *5α-DHT* 5α-dihydrotestosterone, *DHEAS* dehydroepiandrosterone-sulfate, *HR* hazard ratio, *SHBG* sex hormone binding globulin. Data are HRs for 1 standard deviation change in each biomarker based on cox regression analyses.

^a^Adjusted for age, education, BMI, smoking, alcohol use, number of treated chronic diseases, oral contraceptives, hormonal replacement therapy use, and menopausal status. The models for time to first or recurrent MDD were also adjusted for lifetime MDD or anxiety, and models for time to remission of MDD were additionally adjusted for lifetime anxiety disorder.

### Mediation analysis

Free testosterone levels were positively associated with salivary cortisol levels, though only significant for AUC_g_ (*p* = 0.0006) but not for AUC_i_ (*p* = 0.09). However, neither AUCi nor AUCg mediated the association between free testosterone and MDD.

## Discussion

In this study, we examined the cross-sectional and longitudinal association between androgens and the presence and course of MDD in pre- and postmenopausal women aged 18 to 65. In contrast to our first hypothesis, only plasma free-testosterone levels were significantly higher in women with current MDD compared to never MDD, but this difference was not large enough to be considered clinically meaningful (statistically equivalent). Still, this group difference was independent of important confounders such as age and oral contraceptive use, and was not explained by hyperactivity of the HPA axis. All other differences in plasma androgen and SHBG levels between women with current or remitted MDD and never MDD were not statistically significant and also statistically equivalent. Moreover, none of the androgens predicted the time to a new MDD during 4 years of follow-up. Hence, the general negative findings of this study, suggest that plasma androgens do not play an important role in the pathogenesis of depressive disorders in women.

Our finding that women with current MDD had higher levels of free testosterone than women with never MDD differs from results from previous studies which found similar levels for depressed and non-depressed women^13,15,17^. However, previous studies used radioimmunoassays rather than LC-MS/MS^13,17^, or lifetime rather than current MDD for group definition^15^, and hence may have lacked sensitivity to detect subtle differences. Yet, in contrast to free testosterone, total testosterone levels rendered significance after correction for multiple testing and covariates. This finding is in line with two studies in 215 and 634 women showing no significant differences in total testosterone levels between women with and without MDD^12,13^. Still, a large previous study in 3302 women revealed that higher testosterone levels were slightly more common among depressed women compared to non-depressed controls^9,17^. However, this finding would not have remained statistically significant if a multiple testing correction would have been performed. Therefore, the overall non-significant differences in androgen levels between women with and without MDD after the adjustment for covariates, underscore the importance of the execution of such sensitivity analyses.

Interestingly, free testosterone but none of the other androgens were associated with MDD. This may be explained by the fact that other androgens, in contrast to free testosterone, have a higher binding affinity to SHBG and/or albumin^30^. Hence, free testosterone is the only androgen that can freely pass through the blood–brain barrier. In support of this hypothesis, 5α-DHT has a stronger affinity to binding proteins than total testosterone and androstenedione, and indeed the associations between the latter two and MDD were stronger than for 5α-DHT. Once free testosterone has entered the brain, there are a myriad of mechanisms by which it may affect mood. It can for example bind to androgen receptors in brain regions involved in affect regulation^31,32^. Free testosterone can also interact with other important regulatory systems implicated in mental illnesses, such as neurotransmitter and immune systems^31,33–35^. On the other hand, free testosterone might also be altered as a consequence of MDD, for example, due to a chronic stress response. Although cortisol and free testosterone were positively associated, salivary cortisol did not mediate the association between free testosterone and MDD. If anything, one should bear in mind that although free-testosterone levels were on average higher in women with current MDD compared to women with never MDD, this difference was found to be statistically equivalent. This suggests that, although present, the difference was of no or limited clinical relevance.

In contrast with our second hypothesis, we did not find an association between androgens and incident MDD during 4 years of follow-up. This is in concordance with previous prospective studies which followed 980, 1711, and 3840 adult women from the general population for up to 10 years and showed no association of total testosterone with incidence of MDD^14–16^. Nevertheless, one previous study showed that an annual increase in total testosterone was associated with increased odds of depressive symptoms in perimenopausal women using the self-report CES-D. However, in this study, testosterone was measured with immunoassays, and analyses were not adjusted for lifetime MDD^17^. This is important because lifetime MDD presence is the main predictor for a future episode^36^.

Additionally, in contrast to our third hypothesis, we did not find an association between baseline androgens levels and time to remission. This suggests that though androgens might be associated with a current diagnosis of MDD, it has no or only limited predictive power. Altogether, there is little evidence for a temporal association between single androgen levels within the physiological range and the development of MDD in women, but the current data cannot reject the possibility that changes in androgen levels are of importance to the development of MDD in women as we only measured androgens at baseline. Studying testosterone during windows of development in which it may also cause permanent effects on cerebral functioning, such as antenatally or during puberty, may be an intriguing alternative direction of research^37^.

Strengths of this study include the examination of a well-phenotyped sample where psychiatric diagnoses were based on structured diagnostic interviews (CIDI) and the sample size was principally capable of detecting clinically significant differences between groups. Importantly, we used LC-MS/MS for the determination of total testosterone, androstenedione, and 5α-DHT, which is imperative given the fact that detection of physiologic levels of androgens that are seen in women (e.g., testosterone <5.0 nmol/L) are less reliable when using immunoassay levels due to cross-reactivity with other hormones^19^. Furthermore, we were able to adjust for a wide range of confounders. As confirmed by the differences in androgen levels between oral contraceptive users and non-users in our study, oral contraceptives were associated with lower levels of androgens, especially total testosterone, likely through inhibiting ovarian and adrenal androgen synthesis and by increasing levels of SHBG^38,39^.

Some limitations of our study merit further discussion. First, because we used observational data, we cannot conclude whether elevated free-testosterone levels in MDD are a cause or a consequence of the disease. Second, peripherally measured androgens might lack the sensitivity to reflect androgen levels in the brain. Though all androgens can pass the blood–brain barrier, binding proteins limit the extent. Third, the lack of prospective associations might be because changes in free testosterone were a consequence of MDD rather than a cause, and that single measurements may be insufficient to predict psychopathology over longer time periods. Additionally, androgens have diurnal variation, vary by the phase of the menstrual cycle, and decline with age^40,41^. Nonetheless, all blood draws were done in the morning with little variance, and there was no statistical difference in our findings related to menopausal status or days since the last menstrual cycle of those women not using oral contraceptives and not being menopausal between the psychopathological groups. Moreover, we have adjusted for important covariates. Therefore, strong associations would resist this limitation, suggesting that, if present at all, prospective associations would have been weak. Finally, although the sociodemographic characteristics of the sample with and without follow-up were comparable and the attrition rate was high (13.7%), the sample without follow-up had more depressive symptoms than the sample with follow-up at baseline. This may point to a selection bias for the longitudinal analyses.

In summary, this study showed no differences in most androgen levels between adult women with and without MDD. Higher plasma levels of free testosterone in women with MDD compared to women without a history of MDD were found, independent of important confounders like age and oral contraceptive use. However, these levels did not predict future MDD. Therefore, this study suggests that the role of plasma androgen levels in women’s vulnerability for MDD is limited.

## Supplementary information

Supplements.
