# Supplementary material for: Plasma androgens and the presence and course of depression in a large cohort of women
Source: Transl Psychiatry. 2021 Feb 12;11:124. doi: 10.1038/s41398-021-01249-2 (PMC7881099; doi:10.1038/s41398-021-01249-2)
Supplement: Supplementary file 1 — Supplements. [file 41398_2021_1249_MOESM1_ESM.docx]

**Supplements**

**Supplement 1.** Characteristics of 1432 participants with follow-up and 227 participants without follow-up at baseline.

|  | **Sample with follow-up** | **Sample without follow-up** |  |
| --- | --- | --- | --- |
|  | (n = 1432)^a^ | (n = 227)^a^ | *p* |
| **Age**, mean (SD) | 41.3 ± 13.0 | 39.6 ± 12.8 | .07 |
| **Education**, mean (SD) | 12.4 ± 3.2 | 11.3 ± 3.1 | <.0001 |
| **Body Mass Index**, mean (SD) | 25.2 ± 5.4 | 25.6 ± 5.1 | .31 |
| **Current smoking**, no. (%) | 504 (35.1) | 117 (51.5) | <.0001 |
| **>1 alcohol unit/day**, no. (%) | 185 (12.9) | 28 (12.3) | .91 |
| **Treated chronic diseases**, geo. mean (95%CI) | 0.5 (0.5-0.5) | 0.5 (0.4-0.6) | 0.49 |
| **Oral contraceptive use**, no. (%) | 390 (27.2) | 62 (27.3) | 1.00 |
| **Hormonal replacement therapy use**, no. (%) | 25 (1.7) | 3 (1.3) | 1.00 |
| **Menopausal**, no (%) | 449 (34.2) | 57 (27.5) | .07 |
| **Antidepressant use**, no. (%) | 369 (25.8) | 73 (32.2) | .05 |
| **Current MDD**, no. (%) | 405 (28.3) | 108 (47.6) | <.0001 |
| **Current anxiety disorder**, no. (%) | 499 (34.8) | 124 (54.6) | <.0001 |
|  |  |  |  |
| *Predictors - Androgens* |  |  |  |
| **Total testosterone** (nmol/L), geo. mean (95%CI) | 0.73 (0.71-0.74) | 0.73 (0.69-0.77) | .84 |
| **SHBG (nmol/L)**, geo. mean (95%CI) | 64.2 (62.2-66.2) | 61.4 (56.8-66.4) | .29 |
| **Free testosterone** (pmol/L), geo. mean (95%CI) | 8.08 (7.84-8.33) | 8.35 (7.77-8.96) | .44 |
| **5α-DHT (nmol/L),** geo. mean (95%CI) | 0.32 (0.31-0.33) | 0.34 (0.31-0.36) | .16 |
| **Androstenedione** (nmol/L), geo. mean (95%CI) | 3.07 (2.99-3.16) | 3.27 (3.06-3.49) | .10 |
| **DHEAS** (µmol/L), geo. mean (95%CI) | 4.40 (4.25-4.55) | 4.53 (4.22-4.86) | .49 |

Abbreviations: ANOVA, analysis of variance; BMI, body mass index (calculated 1 as weight in kilograms divided by height in meters squared); MDD, Major Depressive Disorder; 5α-DHT, 5α−dihydrotestosterone; DHEAS, dehydroepiandrosterone-sulphate; SHBG, sex hormone binding globulin.

To convert nmol/L to ng/dl for total t, 5α-DHT, and androstenedione multiply by 28.84, 29.07, and 28.64, respectively. To convert pmol/L to pg/ml for free testosterone divide by 3.47. To convert µmol/L to µg/dl for DHEAS, multiply by 36.85. To convert to nmol/L to μg/ml for SHBG, multiply by 0.095.

^a^ Based on one-way ANOVA for independent samples or χ2 tests.

**Supplement 2 - Motivation for including covariates and their definitions**

Below, we describe the motivation and the definition of the covariates that were used in the analyses.

- *Sociodemographics*

Motivation: Age and education were used as covariates to control for basic sociodemographic differences.

Definition: Both age and education were defined in years.

- *Current smoking status*

Motivation: Current smoking status was added to the models as smoking affects testosterone levels(1), and smokers are more likely to be depressed (see Table 1).

Definition: Smoking status was ascertained with a question whether participants currently smoked or not.

- *Alcohol use (≤ or >7 units a week)*

Motivation: Alcohol use was considered as a covariate as it may increase the risk for depression(2), and chronic use may cause hormonal disturbances(3).

Definition: Alcohol use was ascertained with two questions from the Alcohol Use Disorders Identification Test (AUDIT)(4). By combining the answers from the question on how often a participant drinks, and the question that assessed the typical quantity when a participant drinks, the number of alcohol units per week were calculated. We dichotomized the number of drinks in seven or less and more than seven as use from the first mentioned group would be considered responsible alcohol use following Dutch guidelines(5).

- *Body Mass Index (BMI)*

Motivation: BMI was considered as a covariate as higher testosterone levels are associated with higher measures of BMI(6), and higher measures of BMI are associated with more depressive symptoms(7).

Definition: BMI was defined as weight in kg divided by square of the body height.

- *Number of treated chronic diseases*

Motivation: In men, testosterone levels are affected by health status(8). Although that is unknown in women, we assumed that testosterone levels might be affected by other somatic diseases as well. And as the number of chronic diseases is associated with an increased risk of depressive symptoms(9), this variable was considered a covariate.

Definition: The number of chronic diseases were determined using a 21-item face to face interview instrument that is designed for the NESDA. The respondents were asked if they have a certain specified chronic disease. If a disease is present, respondents were further asked if they used medication and if they were under treatment by a physician for their disease. The following diseases are asked; asthma, chronic bronchitis or pulmonary emphysema; heart diseases or infarct; diabetes; cerebrovascular infarction or hemorrhage; arthritis or arthrosis; rheumatic complaints; benign or malignant tumor & metastasis; high blood pressure; stomach or intestinal ulcer; intestinal disorders; liver disease or liver cirrhosis; epilepsy; chronic fatigue syndrome; allergies; thyroid gland disease; injury (last year); head injury (ever); another chronic disease. In the last item of the questionnaire (item 21) respondents were asked about any other chronic disease that had not previously been mentioned. Respondents could mention up to five additional chronic diseases. For neurologic disorders, a separate variable was constructed including migraine & cluster headache, multiple sclerosis, neuropathy and other neurologic diseases. A count of number of treated chronic diseases was made. All diseases were included for which medications were used or respondents were treated, except allergies, injuries, head injuries, chronic fatigue syndrome, high blood pressure, and angina. Osteoarthrosis and rheumatism were combined and counted as one, since people often confuse these diseases. Regardless of how many other diseases were mentioned in the last question (question 21 - Another chronic disease), this item was counted as one disease. For intestinal disorders some categories were excluded from the count as they were considered to be symptoms instead of pathologies. These categories were diarrhea, obstipation, stress-induced complaints, cramps and irregular defecation.

The number of participants (%) of five prevalent groups of disorders were the following:

- - Lifetime prevalence heart condition or heart attack n = 64 (3.9)
  - Diabetes n = 46 (2.8)
  - Lifetime prevalence stroke n = 23 (1.4)
  - Rheumatoid arthritis n = 21 (1.2)
  - Lifetime prevalence cancer n = 136 (8.2)
- *Current oral contraceptive use*

Motivation: As oral contraceptive use lowers androgen levels by increasing SHBG levels (see Supplementary Table 2) and is associated with an increased risk for depressive symptoms and depression(10, 11), oral contraceptive use as included as a covariate.

Definition: Current oral contraceptive use was ascertained with the question, "Do you take a contraceptive pill?".

- *Hormonal replacement therapy use*

Motivation: As for oral contraceptive use, hormonal replacement therapy use lowers androgen levels and hence should be considered as a covariate(12).

Definition: Current hormonal replacement use was ascertained when women used medications with ATC-code G03CA of G03F.

- *Menopausal status*

Motivation: As androgen production by the ovaries ceases after menopause, being post menopausal affects your androgen levels(13). Also, the risk for depression is most prominent during the reproductive years. Hence, menopausal status was included as a covariate(14).

Definition: Postmenopausal status was determined with the question, "Do you (still) have a menstrual cycle?". Women who answered the question with "Yes" (n=1082; 65.2%) were defined as being premenopausal, women who answered with "No, menopause" (n=462; 27.8%) were considered postmenopausal, and women who answered with "No, hysterectomy" were categorized as premenopausal when they were aged ≤50 (n=36; 2.2%), or as postmenopausal when they were aged ≥51 (n=58; 3.5%). The latter classification was done, as it was unknown whether the hysterectomy had also included a bilateral oophorectomy.

**Table.** F-statistics with p value for each of the covariates with each of the androgen and SHBG levels in the fully adjusted ANCOVA analyses

|  | **Total testosterone** | | **SHBG** | | **Free testosterone** | | **5α-DHT** | | **Androstenedione** | | **DHEAS** | |
| --- | --- | --- | --- | --- | --- | --- | --- | --- | --- | --- | --- | --- |
| *Covariate* | F | *p* | F | *p* | F | *p* | F | *p* | F | *p* | F | *p* |
| Age | 52.7 | <.001 | 78.3 | <.001 | 44.4 | <.001 | 146.0 | <.001 | 154.4 | <.001 | 5.8 | .02 |
| Education | 0.4 | .54 | .2 | .63 | 1.2 | .27 | 0.3 | .59 | 1.5 | .22 | .1 | .76 |
| BMI | 0.7 | .40 | 81.5 | <.001 | 24.8 | <.001 | 8.6 | .003 | 0.2 | .67 | 220.2 | <.001 |
| Smoking status | 27.9 | <.001 | 9.8 | .002 | 33.2 | <.001 | 54.3 | <.001 | 18.9 | <.001 | 7.9 | .005 |
| Alcohol use | 0.1 | .71 | 1.8 | .18 | 0.0 | .91 | 1.6 | .21 | 17.7 | <.001 | 6.0 | .02 |
| Number of treated chronic diseases | 28.3 | <.001 | 10.0 | .002 | 26.6 | <.001 | 14.4 | <.001 | 63.0 | <.001 | 7.3 | .007 |
| Postmenopausal status | 0.2 | .64 | 12.0 | .001 | 20.5 | <.001 | 8.4 | .004 | 3.3 | .07 | 17.1 | <.001 |
| Oral contraceptive use | 67.5 | <.001 | 527.9 | <.001 | 36.3 | <.001 | 198.4 | <.001 | 19.0 | <.001 | 398.6 | <.001 |
| Hormonal replacement therapy use | 0.0 | .90 | 4.4 | .04 | 0.0 | .94 | 1.3 | .25 | 4.4 | .04 | 8.7 | .003 |

Abbreviations: ANCOVA, analysis of covariance; BMI, body mass index; 5α-DHT, 5α dihydrotestosterone; DHEAS, dehydroepiandrosterone-sulphate; SHBG, sex hormone binding globulin.

All covariates were tested at once using ANCOVA analyses.

1. Shi Z, Araujo AB, Martin S, O'Loughlin P, Wittert GA. Longitudinal changes in testosterone over five years in community-dwelling men. J Clin Endocrinol Metab. 2013;98(8):3289-97.

2. Boschloo L, Vogelzangs N, Smit JH, van den Brink W, Veltman DJ, Beekman AT, et al. Comorbidity and risk indicators for alcohol use disorders among persons with anxiety and/or depressive disorders: findings from the Netherlands Study of Depression and Anxiety (NESDA). J Affect Disord. 2011;131(1-3):233-42.

3. Rachdaoui N, Sarkar DK. Effects of alcohol on the endocrine system. Endocrinol Metab Clin North Am. 2013;42(3):593-615.

4. Saunders JB, Aasland OG, Babor TF, de la Fuente JR, Grant M. Development of the Alcohol Use Disorders Identification Test (AUDIT): WHO Collaborative Project on Early Detection of Persons with Harmful Alcohol Consumption--II. Addiction. 1993;88(6):791-804.

5. van Gool W. Richtlijn goede voeding 2015. Den Haag: Gezondheidsraad; 2015.

6. de Wit AE, Giltay EJ, de Boer MK, Bosker FJ, van der Mast RC, Comijs HC, et al. Associations between testosterone and metabolic syndrome in depressed and non-depressed older men and women. Int J Geriatr Psychiatry. 2019;34(3):463-71.

7. Simon GE, Ludman EJ, Linde JA, Operskalski BH, Ichikawa L, Rohde P, et al. Association between obesity and depression in middle-aged women. Gen Hosp Psychiatry. 2008;30(1):32-9.

8. Bhasin S, Brito JP, Cunningham GR, Hayes FJ, Hodis HN, Matsumoto AM, et al. Testosterone Therapy in Men With Hypogonadism: An Endocrine Society Clinical Practice Guideline. J Clin Endocrinol Metab. 2018;103(5):1715-44.

9. Jokela M, García-Velázquez R, Airaksinen J, Gluschkoff K, Kivimäki M, Rosenström T. Chronic diseases and social risk factors in relation to specific symptoms of depression: Evidence from the U.S. national health and nutrition examination surveys. J Affect Disord. 2019;251:242-7.

10. de Wit AE, Booij SH, Giltay EJ, Joffe H, Schoevers RA, Oldehinkel AJ. Association of Use of Oral Contraceptives With Depressive Symptoms Among Adolescents and Young Women. JAMA Psychiatry. 2019.

11. Skovlund CW, Morch LS, Kessing LV, Lidegaard O. Association of Hormonal Contraception With Depression. JAMA psychiatry. 2016.

12. Casson PR, Elkind-Hirsch KE, Buster JE, Hornsby PJ, Carson SA, Snabes MC. Effect of postmenopausal estrogen replacement on circulating androgens. Obstet Gynecol. 1997;90(6):995-8.

13. van der Veen A, van Faassen M, de Jong WHA, van Beek AP, Dijck-Brouwer DAJ, Kema IP. Development and validation of a LC-MS/MS method for the establishment of reference intervals and biological variation for five plasma steroid hormones. Clin Biochem. 2019;68:15-23.

14. Soares CN, Zitek B. Reproductive hormone sensitivity and risk for depression across the female life cycle: a continuum of vulnerability? J Psychiatry Neurosci. 2008;33(4):331-43.

**Supplement 3.** Adjusted mean plasma androgen and SHBG levels in 1659 women using or not using oral contraceptives at baseline.

|  | **Oral contraceptive use** | **No oral contraceptive use** |  |
| --- | --- | --- | --- |
|  | Geo. mean (95%CI) | Geo. mean (95%CI) | *p* |
|  | n = 452 | n = 1207 |  |
| **Total testosterone** (nmol/L) |  |  |  |
| Unadjusted | 0.69 (0.66-0.72) | 0.74 (0.72-0.76) | .0060 |
| Adjusted^a^ | 0.61 (0.58-0.64) | 0.78 (0.76-0.80) | <.0001 |
| **SHBG** (nmol/L) |  |  |  |
| Unadjusted | 104.7 (100.0-109.8) | 53.0 (51.5-54.6) | <.0001 |
| Adjusted^a^ | 98.8 (94.1-103.7) | 54.2 (52.7-55.8) | <.0001 |
| **Free testosterone** (pmol/L) |  |  |  |
| Unadjusted | 5.26 (5.02-5.52) | 9.54 (9.27-9.83) | <.0001 |
| Adjusted^a^ | 4.83 (4.60-5.08) | 9.86 (9.57-10.15) | <.0001 |
| **5α-DHT** (nmol/L) |  |  |  |
| Unadjusted | 0.34 (0.32-0.35) | 0.31 (0.30-0.32) | .01 |
| Adjusted^a^ | 0.28 (0.26-0.29) | 0.34 (0.33-0.35) | <.0001 |
| **Androstenedione** (nmol/L) |  |  |  |
| Unadjusted | 2.90 (2.76-3.05) | 3.17 (3.08-3.27) | .0026 |
| Adjusted^a^ | 2.31 (2.20-2.42) | 3.46 (3.37-3.55) | <.0001 |
| **DHEAS** (µmol/L) |  |  |  |
| Unadjusted | 5.19 (4.89-5.51) | 4.15 (4.01-4.31) | <.0001 |
| Adjusted^a^ | 3.96 (3.75-4.19) | 4.60 (4.45-4.75) | <.0001 |

Abbreviations: ANCOVA, analysis of covariance; 5α-DHT, 5α dihydrotestosterone; DHEAS, dehydroepiandrosterone-sulphate; SHBG, sex hormone binding globulin. To convert nmol/L to ng/dl for total testosterone, 5α-DHT, and androstenedione multiply by 28.84, 29.07, and 28.64, respectively. To convert pmol/L to pg/ml for free testosterone divide by 3.47. To convert µmol/L to µg/dl for DHEAS, multiply by 36.85. To convert to nmol/L to μg/ml for SHBG, multiply by 0.095. Data are geometric means based on two-way ANCOVA analysis.

**^a^** Adjusted for age, education, BMI, smoking, alcohol use, number of treated chronic diseases, menopausal status, hormonal replacement therapy use, and psychopathology

**Supplement 4.** Mean plasma androgen and SHBG levels in 1659 women according to their psychopathology.

|  | **Never MDD** | **Remitted MDD** |  | **Current MDD** |  |
| --- | --- | --- | --- | --- | --- |
|  | Geo. mean (95%CI) | Geo. mean (95%CI) | *p* | Geo. mean (95%CI) | *p* |
|  | n = 392 | n = 754 |  | n = 513 |  |
| **Total testosterone** (nmol/L) |  |  |  |  |  |
| Unadjusted | 0.68 (0.65-0.71) | 0.74 (0.71-0.77) | .005 | 0.74 (0.71-0.78) | .007 |
| Adjusted^a^ | 0.69 (0.66-0.72) | 0.74 (0.71-0.76) | .02 | 0.74 (0.71-0.77) | .02 |
| **SHBG** (nmol/L) |  |  |  |  |  |
| Unadjusted | 67.7 (63.8-71.8) | 64.3 (61.6-67.2) | .18 | 60.3 (57.3-63.6) | .004 |
| Adjusted^a^ | 65.4 (62.3-68.9) | 64.5 (62.3-66.9) | .66 | 61.7 (59.2-64.4) | .09 |
| **Free testosterone** (pmol/L) |  |  |  |  |  |
| Unadjusted | 7.26 (6.86-7.69) | 8.23 (7.89-8.58) | .0006 | 8.66 (8.23-9.12) | <.0001 |
| Adjusted^a^ | 7.55 (7.19-7.92) | 8.17 (7.89-8.47) | .01 | 8.50 (8.13-8.87) | .0005 |
| **5α-DHT** (nmol/L) |  |  |  |  |  |
| Unadjusted | 0.31 (0.30-0.33) | 0.32 (0.31-0.33) | .60 | 0.33 (0.31-0.34) | .26 |
| Adjusted^a^ | 0.32 (0.30-0.33) | 0.32 (0.31-0.33) | .90 | 0.32 (0.31-0.34) | .48 |
| **Androstenedione** (nmol/L) |  |  |  |  |  |
| Unadjusted | 2.88 (2.73-3.04) | 3.11 (2.99-3.23) | .03 | 3.26 (3.11-3.41) | .0007 |
| Adjusted^a^ | 2.97 (2.83-3.10) | 3.10 (3.00-3.20) | .13 | 3.20 (3.07-3.33) | .02 |
| **DHEAS** (µmol/L) |  |  |  |  |  |
| Unadjusted | 4.55 (4.27-4.85) | 4.36 (4.16-4.57) | .29 | 4.39 (4.15-4.65) | .41 |
| Adjusted^a^ | 4.56 (4.32-4.82) | 4.38 (4.22-4.56) | .23 | 4.35 (4.15-4.56) | .20 |

Abbreviations: ANCOVA, analysis of covariance; 5α-DHT, 5α dihydrotestosterone; DHEAS, dehydroepiandrosterone-sulphate; MDD, Major Depressive Disorder; SHBG, sex hormone binding globulin. To convert nmol/L to ng/dl for total testosterone, 5α-DHT, and androstenedione multiply by 28.84, 29.07, and 28.64, respectively. To convert pmol/L to pg/ml for free testosterone divide by 3.47. To convert µmol/L to µg/dl for DHEAS, multiply by 36.85. To convert to nmol/L to μg/ml for SHBG, multiply by 0.095. Data are geometric means based on two-way ANCOVA analysis with women who never experienced a MDD as the reference group.

**^a^** Adjusted for age, education, BMI, smoking, alcohol use, number of treated chronic diseases, menopausal status, oral contraceptive use, and hormonal replacement therapy use.

**Supplement 5.** Data distribution of androgen and SHBG levels in women according to their diagnosis at baseline.


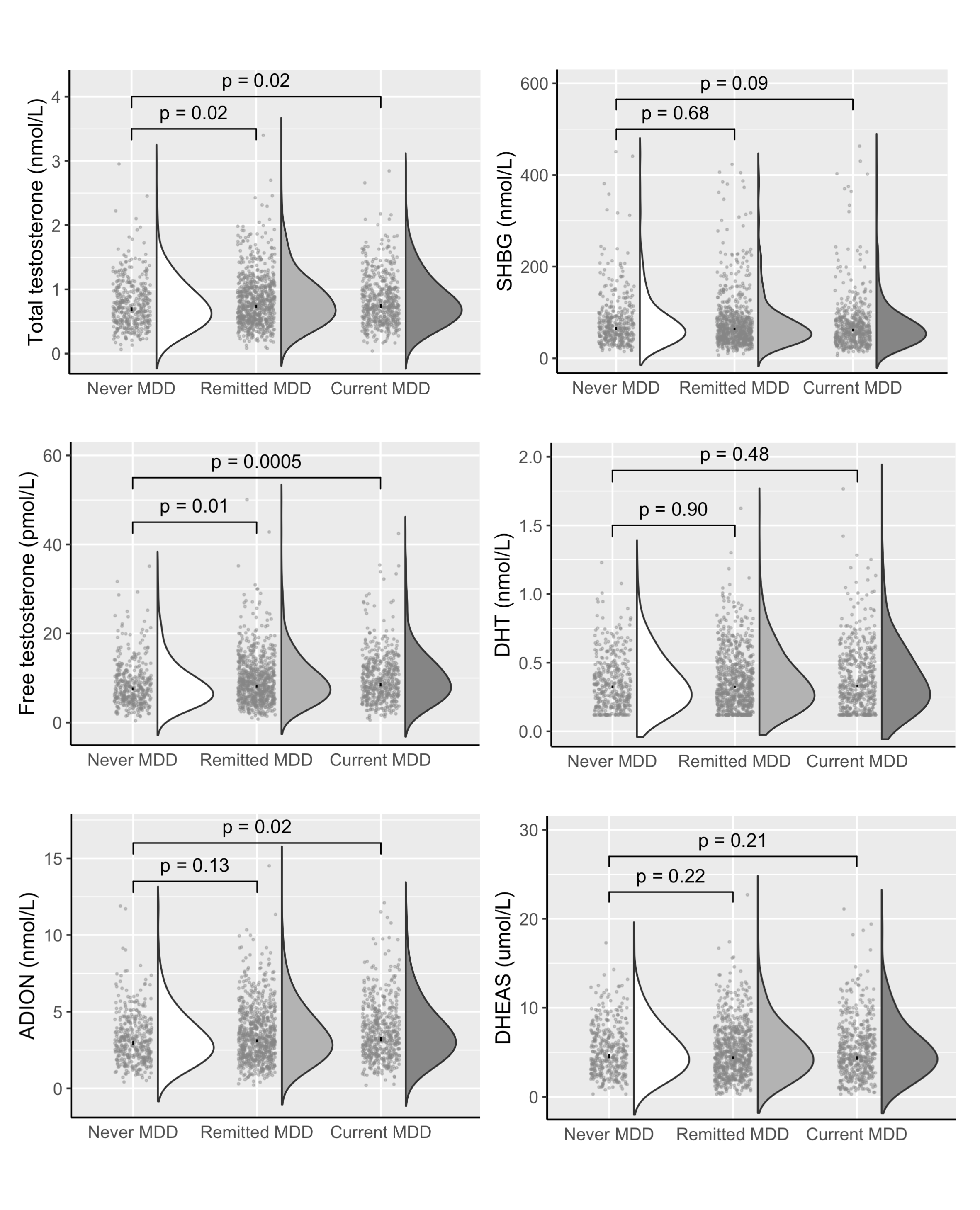


Abbreviations: DHT, 5α dihydrotestosterone; DHEAS, dehydroepiandrosterone-sulphate.

Figures are raincloud plots that combines an illustration of the data distribution with jittered raw data. The black lines in the jittered raw data are the 95% CI of the geometric means (back transformed means of log transformed data). P-values are based on adjusted mean analyses by ANCOVA models. Models were adjusted for age, education, BMI, smoking, alcohol use, number of treated chronic diseases, menopausal status, oral contraceptive use, and hormonal replacement therapy use.

**Supplement 6.** Cross-sectional associations of plasma androgen and SHBG levels and severity of depressive symptoms in 1639 women

|  | **Severity of depressive symptoms** | |
| --- | --- | --- |
|  | β (95%CI) | *p* |
| **Total testosterone** |  |  |
| Unadjusted | 0.006 (-0.003 to 0.014) | .18 |
| Adjusted^a^ | 0.008 (-0.001 to 0.016) | .08 |
| **SHBG** |  |  |
| Unadjusted | -0.012 (-0.020 to -0.003) | .007 |
| Adjusted^a^ | -0.004 (-0.015 to 0.006) | .38 |
| **Free testosterone** |  |  |
| Unadjusted | 0.013 (0.005 to 0.022) | .002 |
| Adjusted^a^ | 0.011 (0.001 to 0.020) | .03 |
| **5α-DHT** |  |  |
| Unadjusted | 0.002 (-0.006 to 0.010) | .63 |
| Adjusted^a^ | 0.006 (-0.003 to 0.015) | .17 |
| **Androstenedione** |  |  |
| Unadjusted | 0.006 (-0.003 to 0.014) | .18 |
| Adjusted^a^ | 0.006 (-0.003 to 0.016) | .21 |
| **DHEAS** |  |  |
| Unadjusted | -0.009 (-0.018 to -0.001) | .03 |
| Adjusted^a^ | -0.008 (-0.017 to 0.002) | .12 |

Abbreviations: β, beta coefficient; 5α-DHT, 5α−dihydrotestosterone; DHEAS, dehydroepiandrosterone-sulphate; SHBG, sex hormone binding globulin. Data are β coefficients for 1 standard deviation change in each biomarker based on linear regression models.

**^a^** Adjusted for age, level of education, BMI, smoking, alcohol use, number of treated chronic diseases, menopausal status, oral contraceptive use, and hormonal replacement therapy use.

**Supplement 7.** Equivalence testing for mean plasma androgen and SHBG levels in 1659 women according to their psychopathology.

|  | **Remitted MDD vs. Never MDD** | | | **Current MDD vs. Never MDD** | | |
| --- | --- | --- | --- | --- | --- | --- |
|  | *Mean difference*  *(99.25%CI)* | *SESOI* | *p for equivalence* | *Mean difference*  *(99.25%CI)* | *SESOI* | *p for equivalence* |
|  |  |  |  |  |  |  |
| **Total testosterone** (nmol/L) | -0.05 (-0.10, 0.00) | 0.15 | <.001 | -0.05 (-0.11, 0.01) | 0.17 | <.001 |
| **SHBG** (nmol/L) | 1.00 (-4.50, 6.50) | 16.3 | <.001 | 3.10 (-2.60, 8.80) | 14.0 | <.001 |
| **Free testosterone** (pmol/L) | -0.62 (-1.26, 0.02) | 1.97 | <.001 | -0.95 (-1.66, -0.24) | 1.80 | <.001 |
| **5α-DHT** (nmol/L) | 0.00 (-0.02, 0.02) | 0.07 | <.001 | 0.00 (-0.03, 0.03) | 0.07 | <.001 |
| **Androstenedione** (nmol/L) | -0.13 (-0.36, 0.10) | 0.70 | <.001 | -0.23 (-0.49, 0.03) | 0.64 | <.001 |
| **DHEAS** (µmol/L) | 0.18 (-0.25, 0.61) | 1.23 | <.001 | 0.21 (-0.24, 0.66) | 1.10 | <.001 |

Abbreviations: 5α-DHT, 5α dihydrotestosterone; CI, confidence interval; DHEAS, dehydroepiandrosterone-sulphate; MDD, Major Depressive Disorder; SESOI, smallest effect size of interest; SHBG, sex hormone binding globulin. The mean difference with 99.25%CI was based on the difference between adjusted mean of the two groups that were compared. The 99.25% range was used since cut-off for significance p < 0.0075. For the actual means per group we refer to Supplementary Table 4. The SESOI was calculated on 0.5 * the pooled standard deviation of the baseline levels of the two groups analysed.
